# Development of Schizophrenia in an Autistic Patient With a Rare Chromosome 8p23.1 Deletion

**DOI:** 10.7759/cureus.85045

**Published:** 2025-05-29

**Authors:** Ambria M Pogue, Davin Agustines

**Affiliations:** 1 Medicine, Western University of Health Sciences, Pomona, USA; 2 Psychiatry, Olive View-University of California Los Angeles Medical Center, Los Angeles, USA

**Keywords:** autism spectrum disorder, chromosomal deletion, mental health, neuropsychiatric disorders, schizophrenia

## Abstract

Schizophrenia is a complex disorder influenced by a combination of genetic and non-genetic factors that contribute to its development. Individuals with early neurodevelopmental conditions, such as autism spectrum disorder (ASD), may be at a higher risk for the manifestation of a psychotic disorder later in life. This case describes a female in her mid-20s with a history of ASD, diagnosed in early childhood following delays in developmental milestones and significant academic challenges. Genetic evaluation at that time identified a chromosome 8p23.1 deletion, which was not observed in other family members, consistent with a de novo mutation. Despite having this diagnosis, the patient was able to graduate from high school while being in a mix of special education and partial mainstream programs. However, over the past year, she developed progressive psychotic symptoms characterized by persecutory delusions and behavioral dysregulation, resulting in psychiatric hospitalization and a subsequent diagnosis of schizophrenia. While it is unclear what caused these psychotic symptoms to come about, the neurodevelopmental delays that are attributed to the chromosome 8p23.1 deletion may play a role in not only the development of schizophrenia in this patient but also the severity. This case demonstrates the potential implications of neurodevelopmental and psychotic disorders and highlights the importance of long-term monitoring in individuals with multiple neuropsychiatric conditions.

## Introduction

Schizophrenia is a multifactorial disease where several genetic as well as non-genetic factors have been implicated in its development. Several chromosomes have been shown to have genes that may lead to the development of schizophrenia, including the C4 gene on chromosome 6, as well as possible deletions on chromosome 22 [1]. Recently, chromosome 8p has been looked at in the development of neuropsychiatric disorders, such as autism and schizophrenia [2]. Chromosome 8p consists of 484 genes and accounts for about 1.5% of the genome. Of these genes, 41 have been linked to cerebral development and function. That being said, on the distal portion of chromosome 8p, there are 15 megabases that leave this region susceptible to a high mutation rate [2]. 8p is also a hotspot for copy number variants (CNVs). CNVs are caused by insertions, deletions, or duplications in the DNA that result in a varying number of copies of that DNA sequence. Specifically on chromosome 8p, there are 343 CNVs spread out over 146 genes. Several of these genes have been associated with the development of several disease processes, including schizophrenia, autism spectrum disorder (ASD), cancer, and Crohn’s disease [2].

Schizophrenia is defined by the Diagnostic and Statistical Manual of Mental Disorders, Fifth Edition (DSM-5) as a chronic mental disorder that combines both positive and negative symptoms, which impair activities of daily living (ADL). To be diagnosed with schizophrenia, a patient must have two or more symptoms persisting for at least one month and continuous over at least six months. Symptoms that qualify a patient for this diagnosis include hallucinations, delusions, disorganized speech, disorganized or catatonic behavior, or negative symptoms. The DSM-5 also outlines that if a patient has a history of ASD, they must have prominent delusions or hallucinations for the diagnosis of schizophrenia to be met [3]. ASD is a neurodevelopmental condition marked by social challenges, restricted behaviors, and sensory sensitivities, which typically emerge in childhood [4]. Individuals with ASD experience a broad spectrum of symptoms with varying levels of functional impairment, including communication difficulties, challenges in forming relationships, emotional regulation issues, and a tendency toward rigid interests or routines. Although the diagnosis of schizophrenia is more specific in those with ASD, studies have demonstrated that patients with ASD are six times more likely to develop schizophrenia in their lifetime [4]. In addition to being more likely to develop schizophrenia, patients with ASD may have a worse prognosis than non-ASD patients with schizophrenia. One study demonstrated that greater functional impairment and worse clinical outcomes were present in patients with schizophrenia when paired with ASD. Interestingly, however, ASD has been shown to be protective against internalized stigma associated with schizophrenia [5].

## Case presentation

In this case study, the patient is a female in her mid-20s with a past medical history of autism secondary to a chromosome 8p23.1 deletion, who presented to our hospital after becoming increasingly agitated and violent toward her family. She was diagnosed with ASD at age five when she was not meeting the developmental milestones for her age and was having difficulty in school. Her family was persistent in figuring out the underlying cause, so they were able to get into a genetics clinic where karyotype testing was done, and the chromosomal abnormality was found. The patient does not have any family history or siblings with this chromosomal deletion, nor with autism, suggesting a de novo mutation. Despite having this diagnosis, the patient was able to graduate from high school while being in a mix of special education and partial mainstream programs. She also participated in behavioral therapy throughout her childhood. However, over the past year, the patient began experiencing worsening symptoms of psychosis, including persecutory delusions and agitated behaviors that caused her family to bring her to the inpatient psychiatric unit. Her family had concerns as she was becoming increasingly violent, and they were unsure if they would be able to control her outbursts. During the past year, she also had another inpatient admission to a psychiatric hospital. She was discharged with psychiatric medications that she was unsure of the names of, but she stopped taking them as she felt she did not need them.

When first arriving at the hospital, the patient was placed on an involuntary hold for danger to others and was gravely disabled, meaning that she was unable to meet basic needs due to her mental health disorder. She was then placed on a 14-day hold, which was determined necessary by a court hearing. Due to continued persecutory delusions despite medications, she was put on an additional 28-day hold to optimize her medication regimen and control her positive symptoms. She was first started on a low dose of risperidone, which was gradually increased to 3 mg/day. She was also started on divalproex sodium 500 mg twice a day and lithium 300 mg twice a day. The patient began experiencing increasing side effects, including hair loss, so she was also placed on benztropine, but the divalproex sodium was ultimately discontinued. She was also given a daily vitamin regimen consisting of thiamine, folate, and a multivitamin. Since the patient also has a history of medication non-compliance, she was offered long-acting risperidone injections, but she refused these. Throughout her inpatient stay, the patient was exposed to several stressors that complicated her case. The probable cause hearing for the 28-day hold was especially stressful for the patient, as she was involved in the case and fought for herself to be released. The judge ultimately ruled she was still gravely disabled, thus extending the hold. Immediately following this decision, the patient showed signs of regression. She had increased persecutory delusions, paranoia, and was in a moderate amount of distress. Another stressor involved a sick family member, which prevented her usual visitors from coming to see her. After this event, she once again had an increase in positive symptoms as well as worsening attitudes toward her family members. Although she ultimately had a significant decrease in her symptoms of psychosis with the optimized medication regimen, she continued to have some paranoid delusions even when discharged home. The neurodevelopmental delays that are attributed to the chromosome 8p23.1 deletion may play a role in not only the development of schizophrenia in this patient, but also the severity.

## Discussion

In this case, the complexity of schizophrenia is met with the patient’s limitations on higher cognitive processing and emotional regulation due to her chromosomal deletion. On their own, schizophrenia and ASD are both challenging mental health disorders to treat [6,7]. Pharmacologic treatment of schizophrenia centers around antagonizing dopamine receptors to treat the positive symptoms of schizophrenia, which are thought to be due to an excess release of pre-synaptic dopamine [8]. Both typical and atypical antipsychotics achieve this goal by targeting D2 receptors, although typical antipsychotics are met with more side effects. In addition, these therapies have several shortcomings when targeting negative symptoms such as impaired memory and mood symptoms [8]. Thus, treating schizophrenia is already a challenging task, made more complex by co-morbid mental health conditions such as autism. ASD also has its own set of complications when attempting pharmacological management. A review of medications used to treat ASD states that atypical antipsychotics can treat agitation in this disorder, methylphenidate can be used for hyperactivity, selective serotonin reuptake inhibitors can help in reducing obsessive-compulsive symptoms, and melatonin can be used to aid with sleep [9]. As made clear in this article, there are several different symptoms in ASD that each can require a different pharmacologic agent. This makes treating ASD especially difficult.

Several stressors during our patient’s hospital stay, including the probable cause hearing and concerns about a sick family member, led to regression in her treatment. This highlights the complex interaction between schizophrenia and ASD. While such regression could be attributed solely to the schizophrenia diagnosis, it is more likely that her combined diagnosis of a chromosome 8p23.1 deletion played a significant role. Research has shown that stress in patients with schizophrenia is closely linked to interpersonal relationships [10]. Although the patient had familial support at home, she perceived her family as being unsupportive or working against her. Similarly, both her schizophrenia and ASD precluded her from forming strong connections with people outside her family. This perceived lack of support likely intensified the impact of these stressors during her hospital stay. Beyond social difficulties, the ASD caused by the chromosomal deletion manifested in this patient as an inability to process emotions, further exacerbating the worsening delusions and positive symptoms of schizophrenia, particularly during stressful situations when she was unable to effectively cope with her emotions.

Although schizophrenia is an intricate disorder with several factors, symptomatic remission can be reached in some patients. This is defined as mild or absent symptoms that do not interfere with a patient’s activities of daily living [11]. Predictors of remission in schizophrenia have been previously described as medication adherence, early symptom improvement, less time in a psychotic episode, low illness severity score, high functioning at baseline, and a better premorbid adjustment [12]. Our patient has previously demonstrated medication non-compliance. In addition, it took several medication adjustments to help control her positive symptoms, and at baseline, her functionality is limited by her chromosomal deletion and ASD diagnosis. Risk of relapse in schizophrenia, specifically episodes of psychosis, has also been studied. Medication noncompliance, prior hospitalization, labile family relationships, poor pre-morbid adjustment, and substance use have been found to be among the greatest predictors of relapse [13]. As stated previously, our patient had several of these risk factors. Thus, the prognosis of this case of schizophrenia complicated by ASD is poor, as various health determinants, including overlapping symptoms, treatment complexities, and cognitive and social impairments, make effective management challenging.

## Conclusions

Although chromosome 8p has been of increasing interest lately in neuropsychiatric disorders, there has been no link between the chromosome 8p23.1 deletion and schizophrenia. As a critical region for cerebral development, chromosome 8p should be investigated further to better understand its potential association with schizophrenia. Additionally, the challenges of medical management in patients with both ASD and schizophrenia remain underexplored, especially when these conditions co-occur. More comprehensive research on pharmacologic treatments for both conditions is essential. This case demonstrates that the presence of an autism spectrum disorder due to the chromosome 8p23.1 deletion likely exacerbates the severity and complexity of schizophrenia.
